# Mobile Health Technology Interventions for Suicide Prevention: Systematic Review

**DOI:** 10.2196/12516

**Published:** 2020-01-15

**Authors:** Ruth Melia, Kady Francis, Emma Hickey, John Bogue, Jim Duggan, Mary O'Sullivan, Karen Young

**Affiliations:** 1 School of Psychology National University of Ireland Galway Galway Ireland; 2 Psychology Department Health Service Executive Mid-West Ennis Ireland; 3 Psychology Department Health Service Executive Mid-West Limerick Ireland; 4 Discipline of Information Technology National University of Ireland Galway Galway Ireland; 5 Insight-Centre Discipline of Information Technology National University of Ireland Galway Galway Ireland

**Keywords:** mHealth, systematic review

## Abstract

**Background:**

Digital interventions are proposed as one way by which effective treatments for self-harm and suicidal ideation may be improved and their scalability enhanced. Mobile devices offer a potentially powerful medium to deliver evidence-based interventions with greater specificity to the individual when the intervention is needed. The recent proliferation of publicly available mobile apps designed for suicide prevention underlines the need for robust evidence to promote safe practice.

**Objective:**

This review aimed to examine the effectiveness of currently available mobile health (mHealth) technology tools in reducing suicide-specific outcomes.

**Methods:**

The following databases were searched: Cochrane Central Register of Controlled Trials (The Cochrane Library), MEDLINE, EMBASE, PsycINFO, and relevant sources of gray literature. All published and unpublished randomized controlled trials (RCTs), pseudo-RCTs, and pre-post observational studies that evaluated the effectiveness of mHealth technology in suicide prevention delivered via mobile computing and communication technology were included. Studies were included if they measured at least one suicide outcome variable (ie, suicidal ideation, suicidal intent, nonsuicidal self-injurious behavior, and suicidal behavior). A total of 2 review authors independently extracted data and assessed study suitability, in accordance with the Cochrane Collaboration Risk of Bias Tool, on July 31, 2018. Owing to the heterogeneity of outcomes found across studies, results were not amenable for pooled synthesis, and a meta-analysis was not performed. A narrative synthesis of the available research is presented here.

**Results:**

A total of 7 studies met criteria for inclusion . Four published articles that reported on the effectiveness of the following mobile phone apps were included: iBobbly, Virtual Hope Box, BlueIce, and Therapeutic Evaluative Conditioning. Results demonstrated some positive impacts for individuals at elevated risk of suicide or self-harm, including reductions in depression, psychological distress, and self-harm and increases in coping self-efficacy. None of the apps evaluated demonstrated the ability to significantly decrease suicidal ideation compared with a control condition. In addition, 3 unpublished and recently completed trials also met criteria for inclusion in the review.

**Conclusions:**

Further research is needed to evaluate the efficacy of stand-alone mHealth technology–based interventions in suicide prevention. The small number of studies reported in this review tentatively indicate that such tools may have a positive impact on suicide-specific outcomes. Future mHealth intervention evaluations would benefit from addressing the following 3 main methodological limitations : (1) heterogeneity of outcomes: a lack of standardized measurement of suicide outcomes across studies; (2) ecological validity: the tendency to exclude potential participants because of the elevated suicide risk may reduce generalizability within clinical settings; and (3) app regulation and definition: the lack of a standardized classification system for mHealth intervention type points to the need for better definition of the scope of such technologies to promote safe practice.

**Trial Registration:**

PROSPERO CRD42017072899; https://www.crd.york.ac.uk/prospero/display_record.php?RecordID=72899

**International Registered Report Identifier (IRRID):**

RR2-10.2196/resprot.8635

## Introduction

### Background

#### Suicide Prevention

More than 800,000 people die by suicide every year globally, accounting for 1.4% of all deaths worldwide [1]. Suicide occurs throughout the lifespan and was the second leading cause of death among people aged 15 to 29 years globally in 2012. In addition, it is estimated that 25 suicide attempts (100-200 for youth) occur for every death by suicide [2], resulting in more than 400,000 emergency department visits annually in the United States [3]. Prior suicidal behavior increases the risk of subsequent death by suicide 10- to 60-fold [4-7]. Adolescents with depressive disorders and a history of suicidal behavior are a particularly high-risk group for repeated suicide attempts and suicide [8]. Prospective studies have attempted to predict which individuals will attempt or die by suicide [9], and a diverse range of risk factors that correlate with suicidal behavior has been proposed to support the identification of those at elevated risk, such as sleep disturbances [10], emotion regulation deficits [11], family history of suicide [12], and chronic pain and illness [13].

In a meta-analysis of studies that have attempted to longitudinally predict suicidal thoughts or behavior-related outcomes, Franklin et al [14] found that prediction was only slightly better than chance for all outcomes, and they highlighted several fundamental changes required in future research. They point toward the proliferation of mobile health (mHealth) technologies as a means by which to capture large datasets and to support the expansion of the research base from a focus on risk *factors* to risk *algorithms*. Furthermore, in an attempt to improve the accuracy of suicide estimates, Kristoufek et al [15] found that estimates drawing on Google search data are significantly better than estimates using previous suicide data alone.

In parallel, suicidology researchers have argued that Ecological Momentary Assessment (EMA)—high-frequency data collection in an individual’s usual environment—provides the potential for the development of temporal, individualized prediction of risk states. Thompson et al [16] tested the ability of EMA to predict individual symptom change in suicidal ideation in a sample of 35 adults diagnosed with interepisode bipolar disorder. The results showed that EMA with functional linear models substantially increased the accuracy of prediction of study-emergent suicidal ideation. Advances in mHealth technologies provide potential opportunities to operationalize EMA research to support the sensitive and timely identification of those at risk of suicide.

#### Mobile Health and Suicide Prevention

mHealth is a component of electronic health (eHealth). The Global Observatory for eHealth defines mHealth as “medical and public health practice supported by mobile devices, such as mobile phones, patient monitoring devices, personal digital assistants (PDAs) and other wireless devices” [17]. According to the World Health Organization (WHO), “mHealth involves the use and capitalization on a mobile phone’s core utility of voice and short messaging service (SMS) as well as more complex functionalities and applications including general packet radio service (GPRS), third and fourth generation mobile telecommunications (3G and 4G systems), global positioning system (GPS), and Bluetooth® technology” [18]. mHealth programs and interventions use mHealth technology for a range of functions from data collection tools for health care professionals and clinical decision support systems to supporting health behavior change and disease management by patients in the community.

Although effective face-to-face treatments for self-harm and suicidal ideation are available [19-21], access to effective psychotherapeutic options can be limited. Indeed, negative associations have been found between the availability of mental health services per capita and suicide rates in a number of countries [22]. Stigma and geographical isolation are 2 of the major barriers to help seeking for individuals at risk of suicide [23]. Recent advances in mHealth technology could address these barriers by directing individuals at risk of suicide, who would not otherwise seek help, to access appropriate evidence-based online programs or traditional mental health services [24]. The use of digital technology has been found to be beneficial in the delivery of Web-based suicide prevention interventions [25]. Furthermore, a survey in a psychiatric outpatient setting reported that 69% of respondents and 80% of those aged 45 years or younger indicated a desire to use a mobile app to track their mental health [26]. Public health services are being encouraged to harness digital technology to enhance and support psychological health [27,28]. Digital interventions, in general, have been proposed as one way by which the scalability of effective treatments for self-harm and suicidal ideation may be improved [29,30].

However, the regulation of such technologies and the identification of boundaries in terms of their clinical utility require further attention [31]. In the United States, the Food and Drug Administration (FDA) has announced a precertification program, where app makers are preapproved to release FDA-approved health apps based on fulfilling certain quality control criteria. With more than 250,000 mHealth apps currently available, at least 10,000 of which target mental health conditions [32], the development of this technology is occurring at a faster rate than the evidence base needed to inform it. Mental health was the most common focus of disease-specific mobile apps in 2015, constituting 29% of all chronic condition management. Recent meta-analyses of apps aimed at the management of depression [33], anxiety disorders [34], and self-harm [30] report similar results, with a small evidence base derived from often heterogeneous pilot studies. With thousands of mental health apps readily available through Apple or Google marketplaces, finding a useful tool supported by robust evidence presents a considerable challenge to the individual at risk [35] and to clinicians wishing to use such technology as part of their work.

Despite the motivation to use mHealth technologies, there is a dearth of specific outcomes data on the efficacy of mHealth technology interventions on suicide outcomes. In 2014, Christensen et al [23] conducted a review of the literature on eHealth and suicide. Most eHealth interventions identified in their search were Web based as opposed to mobile based. The researchers concluded that there is some evidence to suggest that suicide interventions via the Web may be effective, but only if they target suicidal content specifically, as opposed to the associated symptoms of depression through cognitive behavioral therapy (CBT). Larsen et al [36] carried out a systematic assessment of mobile phone tools for suicide prevention, screening, and reviewing app content. The researchers concluded that many suicide prevention apps were available, some of which provided comprehensive evidence-based support. Apps with potentially harmful content were also identified.

As digital technology may be particularly attractive for young people, it is essential that mHealth technology and mobile phone apps, in particular, are subject to research evaluation [37] and co-designed with people who have lived experience [38]. Donker et al [39] found that mental health apps evaluated in randomized controlled trials (RCTs) were not publicly available, whereas those with no research evidence were publicly available. In addition, mobile apps that presented harmful content were also identified. Perry et al [40] conducted a systematic review of online and mobile psychosocial suicide prevention interventions for adolescents and young adults. The researchers searched 4 major psychological databases for interventions that explicitly targeted suicidality using a mobile, computer, or Web-based app for individuals aged between 12 and 25 years. However, only 1 study met the author’s inclusion criteria. Witt et al [30] reviewed the effectiveness of online and mobile apps (digital interventions) for the self-management of suicidal ideation and self-harm. They identified 14 nonoverlapping studies, the majority of which described online as opposed to mHealth technologies and concluded that overall digital interventions were associated with reductions in suicidal ideation scores at postintervention. There was no treatment effect for self-harm or attempted suicide. Building on the work of Perry et al [40], this review used a broader search strategy to include unpublished studies and ongoing trials. Although Witt et al [30] examined digital interventions more broadly, this review focused on the research evidence examining mHealth technology specifically.

### Why Is It Important to Do This Review?

The objective of this review was to examine the effectiveness of currently available mHealth technology tools in reducing suicide-specific outcomes in individuals taking part in a suicide prevention intervention delivered via mHealth technology.

Following the rise in the number of publicly available mHealth technology tools for suicide prevention, a review of the efficacy of this modality on suicide-specific outcomes is required. A review of the content and usability of available tools has been undertaken [36], and empirical data on their effectiveness in reducing suicide outcomes are warranted.

This review aimed to address the following research question: do suicide prevention mHealth technology tools effectively reduce suicide-specific outcomes?

## Methods

### Overview

The study procedure has been developed in line with the author’s proposed protocol [41], the Preferred Reporting Items for Systematic Review and Meta-Analysis Protocols (PRISMA-P) statement [42], and was registered with the International Prospective Register of Systematic Reviews database (systematic review number: CRD42017072899). In accordance with the PRISMA checklist recommendations, this review used the participants, interventions, comparisons, and outcome(s) (PICO) process for framing and reporting the review criteria; as such, the PICO and study design of the included studies were reported.

### Eligibility Criteria

No restrictions were placed on diagnoses or any clinical or demographic characteristics of eligible samples. Studies using *active* or *inactive* control groups were eligible for inclusion. *Inactive* control groups were those in which participants received no intervention during the trial period (or were placed on a waiting list). *Active* control groups were those that used apps (not aimed at suicide outcomes), face-to-face interventions, or other forms of patient contact to control for the time/attention given to those in the intervention condition. Studies comparing mHealth-based interventions with antidepressants were eligible for inclusion.

### Types of Studies

The types of studies included were all published and unpublished RCTs, pseudo-RCTs, and pre-post observational studies, which evaluated the effectiveness of mHealth technology in suicide prevention. Studies were included if the full report was accessible in English. Only studies that evaluated mobile tools relating specifically to suicide prevention or where suicidality is explicitly mentioned were included.

### Types of Participants

Participants were individuals who took part in a suicide prevention intervention via mHealth technology. No restriction was placed on the age or gender of participants included in the studies reviewed. mHealth technology represents a modality that is accessible across the lifespan. However, the age of participants included in each study was noted in this review, and, where this information was available, it was used to draw conclusions regarding the efficacy of this modality for specific age groups.

### Types of Interventions

Included studies reported on a suicide prevention intervention delivered via mHealth technology, that is, interventions aimed to reduce suicide risk by employing mobile communication or mobile computing technology. The review included studies with psychological and nonpsychological interventions (eg, psychoeducation, diaries, mood monitors, and self-management programs). As defined by Slattery et al [43] in a protocol for a systematic review on eHealth interventions for chronic pain, psychological treatments are those that explicitly deliver a psychological component (eg, psychotherapy for suicidal thoughts). Studies were included regardless of treatment intensity or duration.

### Outcomes

#### Primary Outcomes

Included studies comprised at least one suicide-specific outcome. This could include suicidal behavior, nonsuicidal self-injurious behavior, suicidal ideation, and suicidal intent.

#### Secondary Outcomes

Secondary outcomes were symptoms of depression or anxiety, as measured using administered or self-reported scales, where this information was available.

### Search Strategy

All databases were searched from their start date. Studies were included if a full-text paper in English was made available, either through databases or through contact with the study authors. The following databases were searched: MEDLINE, EMBASE, PsycINFO, and Cochrane Central Register of Controlled Trials (The Cochrane Library). Although the same search strategy was used for each database, appropriate changes were made to accommodate the different interfaces. Details of the search strategy are provided in Textbox 1. Medical Subject Headings and text word terms were used.

Clinical trial registries were searched to identify completed and in-progress trials. This included ClinicalTrials.gov, the metaRegister of controlled trials, and the WHO International Clinical Trials Registry Platform. Grey literature was searched using the OpenGrey database, which included technical or research reports, doctoral dissertations, and conference papers from the previous 5 years.

The reference lists of relevant systematic reviews and of included studies were also searched to identify additional studies that may be relevant. A handsearch of the *Journal of Medical Internet Research* and a separate search in the PubMed database were also conducted to identify any additional relevant studies.

**Details of the search strategy.**

- mobile* OR mobile phone* OR cell* phone* OR mobile health OR m-health OR mhealth OR mobile app* OR mobile technolog* OR text messag* OR smartphone* OR personal digital assist* OR patient monitoring device*
- suicid* OR suicid* gesture* OR suicid* behavio* OR suicid* idea* OR suicid* attempt* OR self?mutilat* OR self?harm* OR self?injur* OR suicid* intent* OR deliberate self?harm* OR deliberate self?injur*
- trials OR randomised controlled trials OR randomized controlled trials

### The Review Team

The review team managed and conducted the review and had experience in systematic review methods, information retrieval, and statistics. Furthermore, 2 independent investigators (KF and EH) judged article eligibility, with any disagreements resolved through discussion with a third reviewer (RM). In addition, 3 researchers were involved so that measures to minimize bias and error were implemented at all stages of the review.

In addition to the review team, a broader research group was consulted at various stages, including individuals (JD, JB, MO’S, and KY) with expertise in the areas of computer science, clinical psychology, and suicide prevention policy.

### Selection of Studies

**Figure 1:**
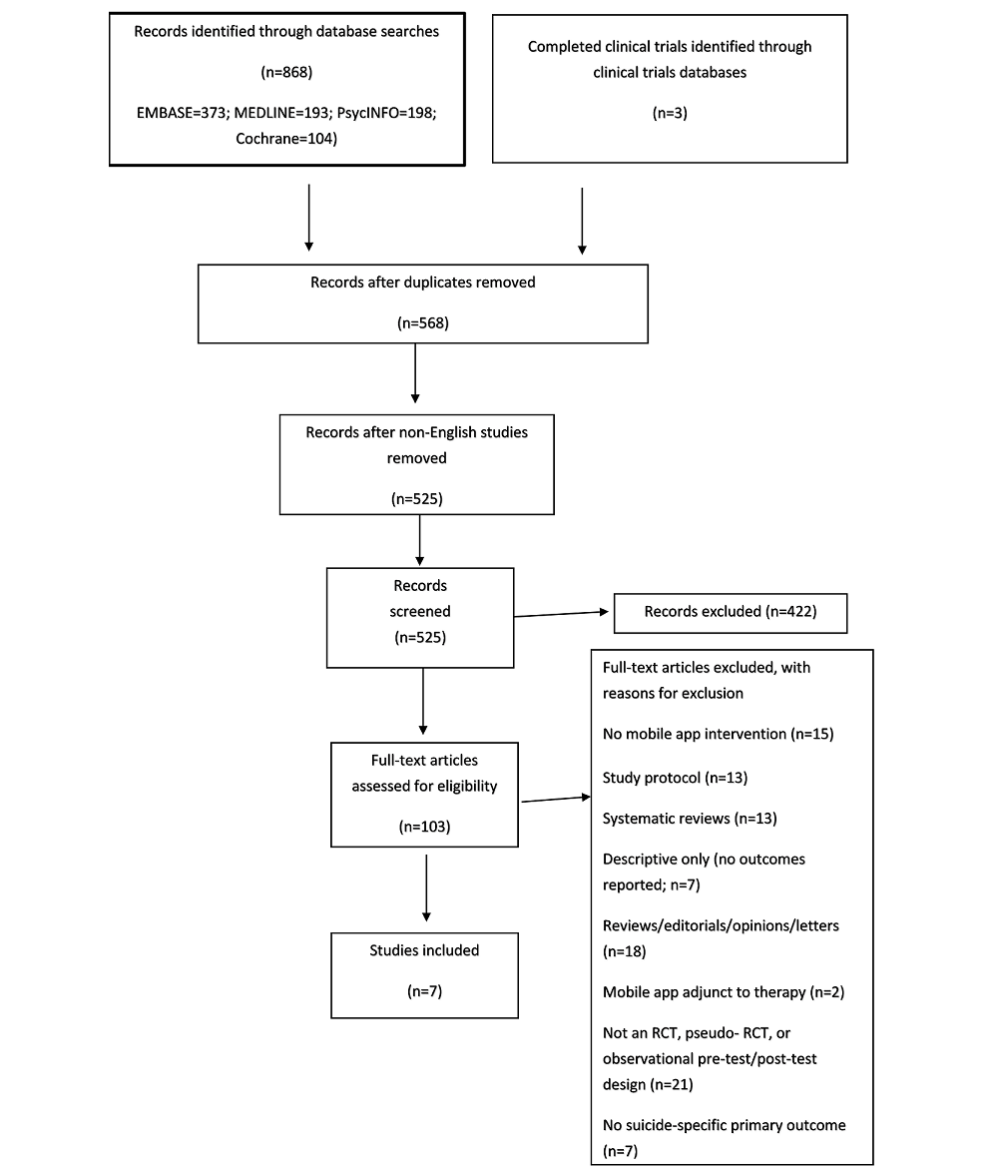
Preferred Reporting Items for Systematic Review and Meta-Analysis flowchart.

Studies that were identified by the search strategy were managed using EndNote X8 [44]. Members of the research team initially screened the titles and abstracts of publications for any duplicates of studies. They then screened for any studies that were not relevant to the review and exported them to a global exclusion folder. All remaining publications were retrieved for further scrutiny. Overall, 2 reviewers independently assessed the full-text articles of the remaining studies for inclusion. Papers that did not meet the inclusion criteria were systematically excluded via the exclusion categories, and the reason for exclusion was recorded. Disagreements between reviewers were discussed with a third reviewer until resolved. A PRISMA flowchart was created to graphically depict the inclusion and exclusion of studies (Figure 1).

### Data Extraction and Management

A data extraction form was created before data extraction. Data were extracted independently by 2 reviewers and verified by another reviewer using a customized form. Where the necessary outcome data were unavailable, the study authors were contacted. The authors were not blind to the study author, institution, or journal. Data were extracted relevant to the following categories: (1) study population and design, (2) intervention, and (3) outcome. Tables were created to present the characteristics of the studies included, containing the following information, where available: participant characteristics, geographic location, assessment periods, assessment/screening measures, description of intervention and comparison interventions, primary and secondary outcomes, theoretical basis, therapeutic content, mode of delivery (mobile phone app, telephone, and text), suicide prevention strategies, behavior change techniques, control condition, intensity and frequency of use, and treatment engagement (retention and attrition).

### Assessment of Risk of Bias in Included Studies

The reviewers independently assessed the risk of bias using the recommended Cochrane Collaboration’s Risk of Bias Tool [45] to assess randomization procedures, bias, allocation, outcome assessor, reporting of findings, and losses to follow-up. Studies were classified as being of low, high, or an unclear risk of bias. The Risk of Bias in Nonrandomized Studies of Interventions was used to assess the risk of bias for controlled before/after designed studies.

### Statistical Methods

The level of heterogeneity was taken into account when considering the suitability of the data for a meta-analysis. A small number of studies were identified with a large amount of heterogeneity present. The data were not amenable to synthesis, and a meta-analysis was not conducted. A full narrative review was undertaken using the *Narrative Synthesis in Systematic Reviews* tool [45].

The narrative synthesis involved the following elements:

- Developing a theory—the theoretical basis of the evaluated interventions was identified.
- Tabulation of data—extracted data from included studies included data on participants, interventions, outcome measures, country of origin, duration, delivery of the intervention, number of participants in each group, context in which intervention was delivered, results, and author comments.
- Textual descriptions summarizing each study were extracted to capture additional findings not identified during earlier stages of the synthesis.
- Vote counting as a descriptive tool—identified studies where the effect of the intervention was positive and statistically significant.

## Results

### Search Results

The PRISMA flowchart of the study selection process is presented in Figure 1. Electronic searches identified 868 publications. The clinical trials database search identified 3 further completed unpublished clinical trials. After excluding duplicates, 568 studies remained. After non-English studies were removed, 525 abstracts and titles were screened for suitability, with 422 records excluded. A total of 103 full-text articles were then assessed for eligibility via a full-text screening.

Any disagreements regarding study eligibility were resolved following consensus discussions between the 3 reviewers (KF, EH, and RM). The lead authors of the 3 unpublished studies were contacted for further information on program design, study design, data analysis, and methodology, as required. Overall, 4 published studies were included in this review (one of which described 3 RCTs), reporting on a total of 624 participants. A summary of the published studies included in this review is presented in Multimedia Appendix 1.

### Study Characteristics

The studies were conducted in the United States [46], Australia [47] and the United Kingdom [48], with 1 paper reporting on 3 separate studies encompassing data from participants based in Canada, the United States, Australia, and Europe [49]. All included studies reported on samples with a current suicide risk or a history of self-harming and suicidal behaviors. The studies reported on veterans [46], indigenous youth in rural Australia [47], and individuals with a recent and severe history of self-injurious thoughts and behaviors recruited from Web forums focused on self-harm [48] and Child and Adolescent Mental Health Services (CAMHS) patients aged 12 to 17 years [47]. Participants in the Franklin study [48] were recruited from online forums (n=12) that focused on discussions of self-injury and related phenomena. Advertisements did not explicitly describe the study as a treatment study. The informed consent form made the treatment-related aspects of the study clear but did not provide details about Therapeutic Evaluative Conditioning (TEC) that would have allowed participants to discern whether or not they were in the active or control group.

A total of 4 studies met the criteria for inclusion in the review, including 2 RCTs [46,49], 1 pilot RCT [46], and 1 open-phase pre-post trial [47]. Although all studies were inclusive of participants with current or previous self-harm or suicidal ideation, some studies excluded participants who were *seriously contemplating or planning a suicide attempt* [47]. Of the 4 studies, 3 included a control group—only 1 provided a control version of the app [48], whereas the other 2 studies provided face-to-face meetings with study staff and safety checks with the same frequency as participants in the intervention condition [46,50].

In terms of therapeutic modalities, app content was informed by acceptance and commitment therapy (ACT) [46], dialectical behavior therapy [47], CBT [47,50], and TEC [48]. Suicide prevention–specific interventions included asking users to identify reasons for living [50], activities to help develop coping skills [46], emotional regulation strategies [46,47], providing emergency contact details [47,48], and developing an action plan [46]. The iBobbly mobile app [46], based on ACT, was the only of the 4 published studies that followed a module-based approach, whereby participants were asked to complete 3 ACT-based modules within a 6-week period. The Virtual Hope Box, BlueIce, and TEC mobile apps encouraged participants to use the app as often as they felt necessary. Participants in 3 of the 4 studies rated the mobile app with high acceptability [46,47] or high frequency of app use [50].

Suicide-specific outcomes were reported in each study. These included suicide ideation [46,50], self-injurious thoughts [48], and self-injurious behavior or self-harm [47,48]. Outcomes and their definitions differed across studies. A diverse range of measures was used to assess suicide-specific outcomes with no 2 studies using the same measure. Bush et al [50] used the Beck Scale for Suicide Ideation (BSS) [49] and the Columbia Suicide Severity Rating Scale (C-SSRS) [51]. Tighe et al [46] used the Depressive Symptom Inventory Suicidality Subscale [52] and the Patient Health Questionnaire (PHQ; item 9 of the PHQ-9 evaluates passive thoughts of death or self-injury within the last 2 weeks) [53]. Franklin [48] used the Self-Injurious Thoughts and Behaviors Interview [54], and Stallard et al [47] used self-reported changes in self-harming behavior.

In terms of efficacy, 2 studies described a statistically significant positive effect of the mobile app intervention on 1 or more suicide outcomes. Franklin’s analyses of 3 studies reported that TEC produced moderate reductions for all self-injurious thoughts and behaviors except suicidal ideation. Reductions were reported for self-cutting episodes (32%-40%), suicide plans (21%-59%), and suicidal behaviors (33%-77%) [48]. TEC effects were not maintained at the 1-month posttreatment follow-up. Self-reported self-harm behavior reduced for 73% of participants in the intervention condition, who had used the BlueIce app over a 12-week period [47]. All of those who reported not self-harming in the 4 weeks before baseline assessment maintained their status and had not self-harmed over the course of the 12-week trial. Of the 26 participants who had self-harmed at baseline, 4 (15%, 4/26) had completely stopped, with a further 15/26 (58%) reporting less frequent acts of self-harm at follow-up. Tighe [47] reported that although pre- and postintervention changes were significant in the iBobbly arm (*t*_58.1_=2.40; *P*=.02), the interaction of intervention arm by time (pre- vs postintervention) was not significant (*t*_57.8_=1.05; *P*=.30). Estimated marginal means show that any difference between change in the 2 arms arose because of a slight but nonsignificant difference (*t*_59.0_=0.84; *P*=.40) in mean baseline status between the 2 arms. Bush [50] measured the presence and intensity of suicidal ideation (BSS and C-SSRS); importance of reasons for living (Brief Reasons for Living Inventory); feelings of thwarted belongingness (Interpersonal Needs Questionnaire); and how unpredictable, uncontrollable, and overloaded individuals found their lives (Perceived Stress Scale). They found no statistically significant advantage of treatment augmented by the Virtual Hope Box app compared with the control condition for any of these outcomes.

All studies reported significant efficacy of the app interventions on secondary outcomes. Bush et al [50] found that Virtual Hope Box users reported a significantly greater ability to cope with unpleasant emotions and thoughts (Coping Self-Efficacy Scale) at 3 time points. Franklin et al [48] reported that the active group (mean −0.05, SD 0.27) showed a significantly smaller drop in positive affect toward self-related words compared with the control group (mean −0.17, SD 0.24; *t*_49_=−1.77; *P*=.04; Cohen *d*=0.47). Diminished aversion toward the self was associated with less self-cutting (B=−2.49; SE=1.10; incidence rate ratio [IRR]=0.08; *P*=.02), nonsuicidal self-injury (B=−.77; SE=0.17; IRR=0.46; *P*=.001), suicidal ideation (B=*−*1.02; SE=0.20; IRR=0.36; *P*=.001), and suicide plans (B=−.92; SE=0.36; IRR=0.40; *P*=.01). Stallard [47] reported a statistically significant mean difference of 4.91 (*t*_31_=2.11; *P*=.04; 95% CI 0.17-9.64) on postuse symptoms of depression (Mood and Feelings Questionnaire) and 13.53 on symptoms of anxiety (Revised Child Anxiety and Depression Scale; *t*_30_=3.76; *P*=.001; 95% CI 6.17-20.90), which was evident across all anxiety subscales. Table 1 provides a summary of published results for each study.

**Table 1:** Summary of results of published studies included.

Study	Summary of results
Tighe et al, 2017 [46]	Pre-post changes on the Depressive Symptom Inventory Suicidality Scale were significant in the iBobbly condition (*t*_58.1_=2.40; *P*=.02), these differences were not significant when compared with the waitlist condition (*t*_57.8_=1.05; *P*=.30). Participants in the iBobbly group showed statistically significant reductions in the Patient Health Questionnaire-9 and Kessler 10-item questionnaire (K10) scores compared with waitlist. No differences were observed between groups on impulsivity. Waitlist participants improved after 6 weeks of app usage.
Bush et al, 2017 [50]	VHB^a^ users reported significantly greater ability to cope with unpleasant emotions and thoughts (Coping Self-Efficacy Scale) at 3 weeks (B=2.41; 95% CI 0.29-4.55) and 12 weeks (B=2.99; 95% CI 0.08-5.90) compared with the control group. No significant advantage was found on other outcome measures for treatment augmented by the VHB.
Franklin et al, 2016 [48]	TEC^b^ produced moderate reductions for all SITBs^c^ except suicide ideation when compared with the control app. Consistent reductions were seen across studies for self-cutting episodes (32%-40%), suicide plans (21%-59%), and suicidal behaviors (33%-77%). Of 3 studies, 2 showed that TEC impacted on its intended treatment targets, and that greater change in these targets was associated with greater SITB reductions. TEC effects were not maintained at the 1-month posttreatment follow-up.
Stallard et al, 2018 [47]	In all, 73% of those who have recently self-harmed reported reductions in self-harming after using BlueIce for 12 weeks. Statistically significant mean difference of 4.91 (*t*_31_=2.11; *P*=.04; 95% CI 0.17-9.64) on postuse symptoms of depression (Mood and Feelings Questionnaire) and 13.53 on symptoms of anxiety (Revised Child Anxiety and Depression Scale; *t*_30_=33333.76; *P*=.001; 95% CI 6.17-20.90) evident across all anxiety subscales.

^a^VHB: Virtual Hope Box.

^b^TEC: Therapeutic Evaluative Conditioning.

^c^SITB: Self-Injurious Thoughts and Behaviour.

### Risk of Bias in Included Studies

Risk of bias in the 4 published studies was evaluated using the Cochrane Collaboration Risk of Bias Tool [55]. Overall, 3 of the studies had high risk, and 1 had unclear risk, with biases most apparent for the domains of participant, clinical personnel, and outcome assessor blinding. Performance and detection bias, therefore, cannot be ruled out. Attrition bias was reported and was an important limitation for studies with small sample sizes [47,50]. Selection bias was apparent in 2 studies; in particular, Franklin et al [48] recruited participants online; therefore, their motivation and ability to engage may not be reflective of those recruited in the community. In the Stallard study [47], participants were identified by their CAMHS clinician for inclusion, and the study did not include a control group. Participants were also paid to complete the study [48]. Performance bias was evident in studies where allocation concealment was not described [50], and personnel were not blind to the intervention allocation [46], or where no method of blinding was reported in the study [48]. Sensitivity of outcome measures was poor in some studies because of a low number of items [46]. The amount of app usage was not specified in some studies, meaning that the level of engagement was not controlled for [48], and only 1 study controlled for the impact of a digital intervention by providing a control version of the app [48]. Table 2 provides a summary of risk of bias for each study.

**Table 2:** Risk of bias in the published studies included.

Study	Risk of bias
Bush et al, 2017 [50]	Small sample size (attrition bias) Authors did not report how the knowledge of allocated condition was blinded to participants and researchers during the study (performance and detection bias)
Tighe et al, 2017 [46]	Owing to the changes in inclusion criteria after the commencement of the trial, one-fourth (26.2%) of the included participants did not meet the criterion for frequency of suicidal thoughts (attrition bias) Participants, clinical personnel, and outcome assessors were not blind to treatment allocation Data on usage were available for 65.6% of the included participants. Of these, 15% did not complete treatment Small sample size Sensitivity of measures was poor because of number of items (performance bias)
Franklin et al, 2016 [48]	Neither participants nor clinical personnel were blind to treatment allocation (performance bias) Participants were recruited online; therefore, motivation and ability to engage were potentially higher than recruitment through the community (selection bias) Participants paid to complete study Amount of app usage not specified—up to the user, level of engagement was not controlled for
Stallard et al, 2018 [47]	Participants were identified by their Child and Adolescent Mental Health Services clinician. Participants themselves decided whether or not to take part (selection bias) No control group present No blinding reported. All participants in the study received an intervention as there was no control condition (performance and detection bias)

### Unpublished Trials

In all, 3 completed but unpublished trials that met the criteria for inclusion are described in further detail in Multimedia Appendix 2 [56-58].

## Discussion

### Principal Findings

However, the evaluation studies included here indicate that mHealth tools show promise for individuals at elevated risk of suicide and self-harm and can be a useful adjunct to face-to-face therapy. This review confirms and updates the findings of Witt et al [30] who identified only 2 studies describing the efficacy of mobile apps, with the majority of digital intervention studies evaluating the effectiveness of online programs, and adds to the review conducted by Larsen et al [36], which identified that 24 apps for the prevention of suicidal behavior were available for download. This review identified 4 published and 3 unpublished studies evaluating the efficacy of mHealth technology in suicide prevention, indicating that the pace of development of such tools is not matched by evaluative research. Byambasuren et al [59] proposed a concept of *prescriptible* mHealth apps that are currently available, proven effective, and preferably stand-alone. The paucity of robust evidence evaluating the efficacy of mHealth interventions on suicide outcomes indicates that there is not yet sufficient research to support the prescribing of stand-alone mHealth tools for suicide prevention.

Overall, the evaluated mHealth technology interventions demonstrated some positive results for individuals at elevated risk of suicide or self-harm, including reductions in depression, psychological distress, and self-harm, and increases in coping self-efficacy. Mobile apps are one mechanism by which the scalability of effective interventions for suicide prevention can be improved [30]. The use of mobile apps has the capacity to increase accessibility to therapeutic interventions for at-risk individuals who may not otherwise seek help. Digital interventions have the potential to address major barriers to help-seeking behaviors, such as geographical location and stigma [23]. They also provide opportunities for evidence-based intervention to be accessed several times a day and at the time when it is most needed. Participants in 3 of the 4 published studies were encouraged to use the mobile app as often as they felt necessary [46,48,49]. The findings revealed positive results for individuals who have decreased help-seeking behaviors [47] and who were at elevated risk of suicide and self-harm [46-49]. The utilization of mobile apps and other digital interventions is in line with the stepped-care treatment approach [30], which offers the least intensive and most accessible intervention, that has the best chance of delivering a positive outcome as the first line of treatment.

However, neither the iBobbly nor Virtual Hope Box or the TEC apps demonstrated the ability to significantly decrease suicidal ideation compared with a control condition. The studies included reported a positive impact on secondary outcomes, such as measures of depression and anxiety. This confirms parallel findings within face-to-face interventions. Insufficient evidence exists to suggest that CBT focusing on mental illness reduces suicidal cognitions and behaviors. CBT specifically focusing on suicidal cognitions and behaviors has been found to be effective [60]. Such interventions are likely to be most effective if they target the prominent risk factors that exist during acute suicidal crises [61]. The findings of this review may be reflective of the relatively small number of key suicide prevention strategies included in the apps described, with content focused instead on symptoms of depression and anxiety. Similarly, Larsen et al [36] found that although all apps evaluated contained at least 1 component that was broadly consistent with known evidence or best practice guidelines, there was limited concordance with high-quality, evidence-based practice.

In terms of clinical utility, suicide prevention mobile apps can be most beneficial for individuals at risk of suicide, particularly those with decreased help-seeking behaviors, when delivered as an adjunct to therapy, and when they deliver suicide-specific interventions. Future researchers could leverage the current and existing research findings by combining the delivery via mobile app of EMA with evidence-based psychological interventions. In-built EMA in suicide prevention apps has the potential to provide individualized time-stamped data spanning biological, social, and psychological variables, alongside behavioral measures of app usage and engagement to facilitate a greater understanding of suicide processes. The current evidence points to the need to focus on more dynamic intervention approaches, such as Just-In-Time Adaptive Interventions (JITAIs).

### Limitations of the Studies Included

A significant limitation found across all studies pertained to the heterogeneity of the outcomes studied. This hampered the extent to which data could be compared and generalized. Diversity in nomenclature continues to challenge research in this area. Although in the United States, distinction is frequently made between nonsuicidal self-injury and suicidal behavior [62], for example, outside of the United States, these terms are yet to receive widespread acceptance [63]. The need for a standardized and shared labeling of suicidal ideation and behaviors has long been recognized. The International Association of Suicide Prevention Nomenclature Special Interest Group has formed a task force to generate an international standardized nomenclature on all terms within the area of suicidology, inclusive of death wishes, assisted suicide, and bereavement, which may render research more comparable around the world.

Similarly, no 2 studies used the same suicide outcome measure in this review. Studies used combinations of suicide-specific measures, single suicide-specific items, including measures assessing mental health more broadly, and self-report. Heterogeneity of outcomes evident across studies may be reflective of difficulties within research and clinical practice in identifying homogenous subgroups of self-harming patients [64]. It is imperative that to leverage previously completed work and maximize the available data, suicide prevention researchers work to develop and use standard practices in relation to suicide outcome measures.

A further methodological issue, which is likely to reduce ecological validity, is the practice of excluding individuals at increased risk of suicide from participation. Importing empirically supported treatments for depressed adolescents or suicidal adolescents may not be appropriate because the trials in which efficacy was established excluded suicidal teens [61]. Similarly, this review identified studies that excluded participants because of current suicide risk [47], a practice that may contribute to a sample bias and reduce generalizability of findings within clinical settings.

The findings highlight broader issues in relation to regulation and policy pertaining to mHealth technology. Some have argued that it is “now critical, even if paradoxical, to draw new boundaries in the seemingly boundless world of digital health” [31]. Indeed, the specific focus and purpose of the mHealth tools being evaluated in this review were not generally apparent without a full-text review and may underline a lack of standard categorization of tools to guide clinicians in their decision to use or recommend an app as part of their work. Torous and Hsin [31] argue that there is an urgent need to unite the potential of digital health with the fundamental ethics of clinical practice and to encourage innovation while protecting both the future of digital health and the trust it requires to engage patients and clinicians. They propose a practical taxonomy accessible to clinicians, comprising 3 categories of digital health use to illustrate these boundaries: (1) treatment and diagnosis, (2) care enhancement, and (3) resources. It is argued that adopting this rubric at the level of clinical judgment in direct patient care may facilitate empowerment of digital health technology by harnessing the utility of decision-guiding frameworks that clinicians already use in practice. Future research would also benefit from following the guidance of established standards of reporting on eHealth evaluations [65].

### Strengths and Limitations of This Review

This review extends the reviews undertaken by Larsen et al [36] and Witt et al [30] by (1) not restricting the modalities reviewed to mobile phone apps and including other mHealth technology–delivered interventions and (2) evaluating efficacy using outcomes research to complement Larsen’s comprehensive assessment of content. However, the small number of studies identified hindered our ability to synthesize data and conduct a meta-analysis. There were not sufficient data to provide a comparison of mobile technology tools across outcome measures such as mobile phone apps, texting, and gaming, which could help identify the most effective modes of delivery. This may be, in part, contributed to by the restrictive inclusion and exclusion criteria employed within this study. For example, 21 studies were excluded for not being an RCT, pseudo-RCT, or observational pre-/posttest design, and an additional 7 studies were excluded for not having a suicide-specific measure as a primary outcome. Therefore, had the inclusion criteria been less stringent, more studies would have been included within the review, which may have allowed for a meta-analysis to be completed. However, the current eligibility criteria were decided on for this review to capture the results of scientifically rigorous studies.

Risk of bias assessment indicated methodological issues across studies, which could be addressed in future research. The small sample sizes across studies and attrition during studies limited the generalization of findings. Blinding procedures were problematic across studies, and the small number of research-validated suicide prevention apps available makes comparisons with similar interventions difficult. These difficulties may signal a need for a shift in focus from traditional research methods investigating unidirectional cause-effect relationships to examining dynamic systems by applying machine learning approaches to big data.

### Conclusions

The 4 completed and published studies included in this review evaluated the iBobbly, Virtual Hope Box, BlueIce, and TEC apps. Together, results evaluating the apps show some positive results for individuals at elevated risk of suicide or self-harm, including reductions in depression, psychological distress, and self-harm and increases in coping self-efficacy. Despite the continued growth in the development and availability of suicide prevention mHealth tools, there is currently limited evaluative research on the efficacy of such tools in reducing suicide-specific outcomes.

However, neither the use of iBobbly nor Virtual Hope Box or the TEC apps demonstrated the ability to significantly decrease suicidal ideation compared with a control condition. Further evaluation studies would benefit from addressing 3 main methodological issues, which arose across studies: (1) heterogeneity of outcomes, (2) exclusion of individuals at higher risk of suicide, and (3) performance biases arising because of blinding procedures. Although the evidence available indicates some progress, the pace of suicide prevention app development needs to be matched by a greater focus on empirically supported mHealth technology–based interventions. Future research endeavors would benefit from leveraging the current findings with existing EMA research to support the development and evaluation of dynamic interventions (such as JITAIs) delivered via mobile apps to provide effective intervention while simultaneously enhancing our understanding of suicide processes.

## Appendix

Multimedia Appendix 1 Published studies included in the review.

Multimedia Appendix 2 Unpublished completed studies identified.

## Abbreviations

ACT: acceptance and commitment therapy
BSS: Beck Scale for Suicide Ideation
C-SSRS: Columbia Suicide Severity Rating Scale
CAMHS: Child and Adolescent Mental Health Services
CBT: cognitive behavioral therapy
eHealth: electronic health
EMA: Ecological Momentary Assessment
FDA: Food and Drug Administration
JITAI: Just-In-Time Adaptive Intervention
mHealth: mobile health
PHQ: Patient Health Questionnaire
PICO: participants, interventions, comparisons, and outcome(s)
PRISMA: Preferred Reporting Items for Systematic Review and Meta-Analysis
RCT: randomized controlled trial
TEC: Therapeutic Evaluative Conditioning
WHO: World Health Organization
