# Supplementary material for: Mobile Health Technology Interventions for Suicide Prevention: Systematic Review
Source: JMIR Mhealth Uhealth. 2020 Jan 15;8(1):e12516. doi: 10.2196/12516 (PMC6996750; doi:10.2196/12516)
Supplement: Multimedia Appendix 1 [file mhealth_v8i1e12516_app1.docx]

| Study | Study design | Country | Intervention (n) | Control (n) | Participant source | Intervention condition | Control condition | Measures |
| --- | --- | --- | --- | --- | --- | --- | --- | --- |
| Bush et al, 2017 [46] | RCT^a^ | The Unites States  Table 1. Published studies included in the review | 58 | 60 | Recruited from VA Portland Health Care System. US service veterans in active treatment, currently expressing suicidal ideation or had expressed suicidal ideation within 3 months before recruitment. | Patients assigned to the VHB^b^ condition met with the clinical coordinator for instructions on using the app on their personalized smartphones and met with their clinician to guide individual tailoring of VHB content and use of VHB for stress management and emotional regulation. Patients then used VHB as needed while away from the clinic. | Those assigned to the control condition also met with the clinical coordinator following enrollment for orientation to the control group’s printed materials. Printed materials addressed stress management and emotional regulation as needed. | Coping Self-Efficacy Scale, Beck Scale for Suicidal Ideation, Brief Reasons for Living Inventory, Interpersonal Needs Questionnaire, Perceived Stress Scale, and Columbia Suicide Severity Rating Scale |
| Tighe et al, 2017 [47] | RCT | Australia | 31 | 30 | Recruited through a community-based suicide prevention organization. All participants identified as aboriginal and/or Torres Strait Islander. All participants reported clinically significant levels of depression (scores of ≥10 on the Patient Health Questionnaire, psychological distress, and scores of ≥25 on the Kessler Psychological Distress Scale), and self-reported suicidal thoughts in the past 2 weeks. | iBobbly: app-based self-guided cognitive behavioral program consisting of 3 modules completed over 6 weeks. Participants were also encouraged to complete self-assessments on functioning, suicidal thinking, mindfulness exercises, self-soothing activities, and cultural engagement activities. | Participants were allocated to a wait-list control condition. | Suicidal ideation: scores on the Depressive Symptoms Inventory Suicidality Subscale |

| **Franklin et al, 2016 [49]** |
| --- |

| Study 1 | RCT | International | 55 | 59 | Recruited from online forums for self-injury. Participants had to self-report 2 or more episodes of self-cutting in past month to be eligible for participation. | Therapeutic Evaluative Conditioning: app-based gaming conditioning program in which self-harm–related stimuli were sequentially paired with adverse stimuli. Participants were encouraged to interact with the program as often as necessary over a 1-month period. | Participants were allocated to an attentional control condition. | Suicidal ideation: scores on the Self-Injurious Thoughts and Behaviors Interview; Self-harm: scores on the Self-Injurious Thoughts and Behaviors Interview. |
| --- | --- | --- | --- | --- | --- | --- | --- | --- |
| Study 2 | RCT | International | 62 | 69 |  |  |  |  |
| Study 3 | RCT | International | 75 | 84 |  |  |  |  |
| Stallard et al, 2018 [48] | Pre-post trial | The United Kingdom | 37 | None | Young people were recruited from specialist CAMHS^c^ outpatient clinics provided by Oxford Health NHS^d^ Foundation Trust; participants were those who were currently self-harming or who had a history of self-harm; participants were excluded if contemplating or planning a suicide attempt, diagnosed with psychosis, or had a significant learning disability, which may impede their ability to use the smartphone app or had been subject to abuse in the past 6 months. | Eligible participants were provided with the BlueIce app and continued to attend face-to-face meetings with their CAMHS clinician; the BlueIce app includes a mood diary, menu of personalized mood-lifting activities, and automatic routing through safety checks to delay or prevent self-harm. | None | Moods and Feelings Questionnaire; Revised, Child Anxiety, and Depression Scale; Strengths and Difficulties Questionnaire; 10-point Likert scale of app acceptability; and self-reported number of self-harm incidences. Post familiarization, participants were asked to rate the app and their own self-harrm behaviors. Responses were rated on a 5-point Likert scale ranging from 1 (definite no) to 5 (definitely). |

^a^RCT: Randomized Controlled Trial.

^b^VHB: Virtual Hope Box.

^c^CAMHS: Child and Adolescent Mental Health Services.

^d^NHS: National Health Service .
