# Supplementary material for: Mobile Health Technology Interventions for Suicide Prevention: Systematic Review
Source: JMIR Mhealth Uhealth. 2020 Jan 15;8(1):e12516. doi: 10.2196/12516 (PMC6996750; doi:10.2196/12516)
Supplement: Multimedia Appendix 2 [file mhealth_v8i1e12516_app2.docx]

Table 4. Unpublished completed studies identified.

| Study | Study design | Country | Intervention (n) | Control (n) | Participant information | Intervention condition | Control condition | Measures |
| --- | --- | --- | --- | --- | --- | --- | --- | --- |
| Kaslow et al, 2016 [67] (trial registration number: NCT02691221 clinicaltrials.gov) | Clinical trial | The United States | 13 participants | | Aged 18-64 years; enrolled as patients seeking behavioral health treatment through the Grady Health System; sought inpatient and/or outpatient treatment following a suicide attempt or ideation; have personal access to a functioning iPhone and service plan; and speak English. | Participants completed daily mood tracking and completion of a minimum of 1 stress management activity per day. Relaxation exercises to choose from included audio files and consisted of guided meditation, energizing breath, guided visualization for relaxation, progressive relaxation, mindfulness meditation body scan, and mindfulness of breathing. | Single group assignment | Columbia Suicide Severity Rating Scale, Positive and Negative Affect Schedule, R-UCLA loneliness Scale, 14-Item Mental Health Continuum Short Form, 12-item Flourishing Scale, Grit scale, adherence to daily mood tracking and coping skills |
| Fonseca et al, 2016 [68] (trial registration number: NCT03032952 clinicaltrials.gov) | RCT^a^ | The United Kingdom | 84 | 84 | Aged 18 years and older, university students, scored 8 or above on the HADS^b^ | Access to *Feel Stress Free* mobile app for 12 weeks. Instructed to use it at least once per week for 15 min for the first 6 weeks, given free access thereafter. | The control group received 1-2 (noninteractive) text messages per week containing general SoS^c^ concepts (eg, “Remember: more strengths are better when times get tough”). | HADS, Patient Health Questionnaire-9 |
| Pisani et al, 2016 [69] (clinical trial registration number: NCT03145363 clinicialtrials.gov) | RCT | The United States | 222 participants randomly assigned to intervention/control group | | High school students aged 13 to 19 years; New York State high school; healthy participants included. | Intervention group received 2-5 interactive text message sequences per week. Messages invited students to reply using keywords and short free text replies. Messages came from a library of strength-based peer quotations (reviewed for safety), psychoeducational interactions, and games designed to promote emotional skills and use of resources. | The control group received 1-2 (noninteractive) text messages per week containing general SoS concepts (eg, “Remember: more strengths are better when times get tough”). | Difficulties with Emotion Regulation Scales, MFQ^d^, help seeking from adults at school, suicidal ideation—one question from the MFQ about suicidal ideation in the past 2 weeks. |

^a^RCT: randomized controlled trials.

^b^HADS: Hospital Anxiety and Depression Scale.

^c^SoS: Sources of Strength.

^d^MFQ: Mood and Feelings Questionnaire.
